# Mouse‐specific up‐regulation of *Ccnb1* expression by miR‐199a‐5p in keratinocyte

**DOI:** 10.1002/2211-5463.12133

**Published:** 2016-10-24

**Authors:** Bong‐Kyu Kim, Injung Kim, Ah‐Reum Lee, Hye‐In Yoo, Sungjoo Kim Yoon

**Affiliations:** ^1^ Department of Medical LifesciencesThe Catholic University of KoreaSeoulKorea

**Keywords:** *Ccnb1*, keratinocyte, MiR‐199a‐5p, miRNA

## Abstract

MicroRNA (miRNA) are a class of single‐stranded, small non‐coding RNA that regulate various biological processes, including skin and hair cycle regulation, by modulating the expression of specific genes at the post‐transcriptional level. Recently, several studies reported that miRNA directly or indirectly up‐regulate target genes. Previously, we performed microarray analysis to identify the target genes of miR‐199a‐5p in a mouse skin keratinocyte cell line and detected more than 200 genes whose expression was significantly increased by miR‐199a‐5p overexpression (> 1.5‐fold). In this study, we further investigated these genes and found that cyclin B1 (*Ccnb1*) expression was positively regulated by miR‐199a‐5p in keratinocyte. Moreover, *Ccnb1* expression was inversely correlated with miR‐199a‐5p expression during the mouse hair cycle. Cell cycle analysis showed that the proportion of cells in S phase was slightly increased, while the proportion of cells in G2/M phase decreased by miR‐199‐5p. Using luciferase assay, we found that the 3′ untranslated region of *Ccnb1* was a direct target of miR‐199a‐5p. We also found that the regulation of *Ccnb1* expression by miR‐199a‐5p is mouse specific. *CCNB1* expression was not affected in the human and monkey cell lines. These results provide a new relationship between *Ccnb1* and miR‐199a‐5p in both mouse keratinocyte and miRNA biology.

**Abbreviations**
*Ccnb1*: cyclin B1
*Krt23*: keratin 23
MiRNA: microRNA
*Mreg*: melanoregulin
qRT‐PCR: quantitative RT‐PCR

MicroRNA (miRNA) is a class of single‐stranded, small noncoding RNA that consist of 20–22 nucleotides in length. Increasing evidences have revealed that miRNA play significant roles in various biological processes including development, differentiation, growth, and metabolism 1. In general, miRNA primarily regulate gene expression at the post‐transcriptional level through degradation or translational repression of transcript. This regulation is mediated by annealing between the seed sequence of the mRNA and the miRNA motif 2. On the other hand, several investigations demonstrated that miRNA are also able to post‐transcriptionally activate gene expression. These studies suggested that the regulation of target genes by miRNA can be selective, and associated with RNP factors and cellular conditions 3.

In recent years, many investigators have studied the roles of miRNA in skin biology using keratinocytes and cancers, and found that miR‐99b inhibits proliferation of human epidermal keratinocytes by down‐regulating IGF‐1R expression, miR‐378b promotes keratinocyte differentiation by targeting NKX3.1 4, 5. miR‐125b induces tumor initiation and promotes malignant progression by repressing differentiation and increasing survival of cancer cells, and miR‐330‐5p inhibits proliferation and migration of keratinocytes by targeting PDIA3 expression 6, 7. These studies suggest that miRNA are important regulators involved in the proliferation, differentiation, and migration of keratinocytes and in skin cancer.

The miRNA‐199a hairpin precursor gene is located on human chromosome 19, and its orthologous gene resides on mouse chromosome 9 8. Previous studies reported that miR‐199a‐5p is involved in the regulation of biological processes in liver, stomach, testis, colon, and skin keratinocytes 9, 10, 11, 12. Recently, we performed microarray analysis to identify the target genes of miR‐199a‐5p in mouse skin keratinocytes and showed that *Bcam* and *Fzd6* are new target genes in keratinocytes and human cutaneous squamous cell carcinoma 12. Among the 393 putative target genes of miR‐199a‐5p, 232 genes were up‐regulated by miR‐199a‐5p overexpression. In the current study, we focused on these up‐regulated genes and found that the expression of the cyclin B1 gene (*Ccnb1*) is up‐regulated by miR‐199a‐5p. Moreover, we found that this regulation is mouse specific. These results reveal a new relationship between *Ccnb1* and miR‐199a‐5p in mouse keratinocytes.

## Materials and methods

### Mice

The BALB/C mice were bred in the barrier system under specific pathogen‐free conditions with regulated light (07:00–19:00 h), temperature (23 ± 1 °C), humidity (50 ± 5%), and ventilation (10–12 times per hour). All animal experiments were approved by the Institutional Animal Care and Use Committee of the Catholic University of Korea. All experiments were carried out in accordance with the guidelines for animal experimentation.

### Cell culture and transfection experiments

PAM212 (mouse keratinocyte), HaCaT (human keratinocyte), Colo320DM, SNU‐C5 (human colorectal cancer cell), Cos‐1 (monkey kidney fibroblast), and 3T3‐L1 (mouse fibroblast) cells were maintained in Dulbecco's Modified Eagle Medium (DMEM) (Invitrogen, Carlsbad, CA, USA) or Roswell Park Memorial Institute‐1640 Medium (Invitrogen) containing 10% FBS with 5% CO_2_ in a 37 °C incubator. For the miR‐199a‐5p overexpression or inhibition, cells were transfected with a miR‐199a‐5p mimic or inhibitor (Dharmacon, Lafayette, CO, USA) using DharmaFECT 1 transfection reagent (Dharmacon) according to the manufacturer's instruction. The negative mimic or inhibitor (Dharmacon) was used for control purposes at the same concentration as the miR‐199a‐5p mimic or inhibitor. After 72 h of incubation, cells were harvested and used for extraction of total RNA or protein.

### MiR‐199a‐5p‐specific quantitative RT‐PCR

Total RNA from mouse dorsal skin was extracted from the cells using the Trizol reagent (Invitrogen) according to the manufacturer's instruction. A Mir‐X^™^ miRNA First‐strand synthesis kit (Clontech, MountainView, CA, USA) was used to synthesize complementary DNA (cDNA) following the manufacturer's protocol. Quantitative RT‐PCR (qRT‐PCR) was performed using miRCURY LNA^™^ MiR‐330‐5p‐specific primer (Exiqon, Vedbaek, Denmark) following the manufacturer's instruction. The relative expression of miR‐199a‐5p was calculated against U6 small nuclear RNA expression using the comparative ΔΔ*C*_t_ method 13.

### RT‐PCR and qRT‐PCR

Total RNA were reverse‐transcribed into cDNA using a PrimeScript 1st strand cDNA Synthesis kit (Takara, Tokyo, Japan) following the manufacturer's protocol. Thermal Cycler‐100 (MJ Research, Waltham, MA, USA) and CFX96 (Bio‐Rad Laboratories, Hercules, CA, USA) were used to perform RT‐PCR and qRT‐PCR, respectively. The primer sequences and cycling conditions used are listed in Table 1. The relative expression levels were normalized against glyceraldehyde‐3‐phosphate dehydrogenase gene expression using the comparative ΔΔ*C*_t_ method 13. Results represent the average of three independent experiments measured in duplicate.

**Table 1:** List of gene‐specific primers for real‐time PCR

Genes	Accession number	Sequences	Size (bp)	Tm (°C)
*Mreg*	NM_001005423	F: tcagcagaccaaagactcaga	163	60
		R: ggtgctgagtttggtcactg		
*Krt23*	NM_033373	F: cttgccgagtgacttcaagg	296	60
		R: ctgtcagcatgttttccaaagc		
*Ccnb1*	NM_172301	F: ataatccctctccaagcccg	299	60
		R: ggtctcctgaagcagcctaa		
*Cdk1*	NM_007659	F: agagtcactggccagatagt	235	60
		R: aatccatgaactgcccagga		
*CCNB1* (Human)	NM_031966	F: tgaggaagagcaagcagtca	216	60
		R: aacatggcagtgacaccaac		
*CCNB1* (Monkey)	NM_001261149	F: ggccaaaatgcctatgaaga	216	60
		R: gggcttggagagggagtatc		
*Gapdh*	NM_008084	F: aactttggcattgtggaagg	223	60
		R: acacattgggggtaggaaca		
*GAPDH* (Human)	NM_002046	F: gagtcaacggatttggtcgt	238	60
		R: ttgattttggagggatctcg		
*GAPDH* (Monkey)	NM_001195426	F: cgagatccctccaaaatcaa	205	60
		R: tgacgatcttgaggctgttg		

### Western blot analysis

Protein extracts from PAM212 cells were prepared from plates 72 h post transfection using radioimmunoprecipitation assay buffer (150 mm sodium chloride, 1% NP‐40, 0.5% sodium deoxycholate, 0.1% SDS, 50 mm Tris‐HCl [pH 8.0]) according to the standard method. Then, lysates were subjected to 10% SDS/PAGE and transferred to a nitrocellulose membrane. The membrane was incubated with a rabbit polyclonal Ccnb1 antibody (1 : 2500; Santa Cruz, Santa Cruz, CA, USA) or a mouse polyclonal β‐Actin antibody (1 : 5000; Santa Cruz) following the standard protocol. Protein bands were detected using an enhanced chemiluminescence system (Amersham Bioscience, Piscataway, NJ, USA).

### Plasmid construction

The full‐length 3′ UTR cDNA of melanoregulin (*Mreg*), keratin 23 (*Krt23*), and *Ccnb1* was amplified from cDNA generated from the total RNA of PAM212 cells by PCR using PrimeSTAR DNA Polymerase (Takara). The PCR product was cloned into pGEMT‐easy vectors and subcloning into psiCHECK‐2 vector DNA using the *Not*I cloning sites (Promega, Madison, WI, USA).

### Luciferase reporter assay

PAM212 cells (5 × 10^5^/dish) were seeded onto 60‐mm dishes at 70% confluency. After 24 h, cells were transfected into cells with miR‐199a‐5p mimic or control mimic with the reporter construct containing the 3′ UTR of *Mreg*,* Krt23*,* Mcm5* and *Ccnb1* using the Lipofectamine 2000 reagent. Luciferase activity was measured at 48 h post transfection using the Dual‐Luciferase Reporter Assay reagent (Promega).

### Cell cycle assay

MiR‐199a‐5p overexpressed PAM212 cells were harvested at 72 h post transfection and washed with PBS twice. Then, these cells were fixed in 70% ethanol at −20 °C overnight. After washed with PBS, cells were resuspended in propidium iodide staining solution (40 μg·mL^−1^). The percentage of cells in each phase of the cell cycle was measured by FACSCanto II (BD Biosciences, San Jose, CA, USA).

### Statistical analysis

*P* values were determined using Student's *t*‐tests and a value of *P* < 0.05 was considered statistically significant.

## Results

### *Ccnb1* is a target of miR‐199a‐5p in mouse keratinocyte

**Figure 1:**
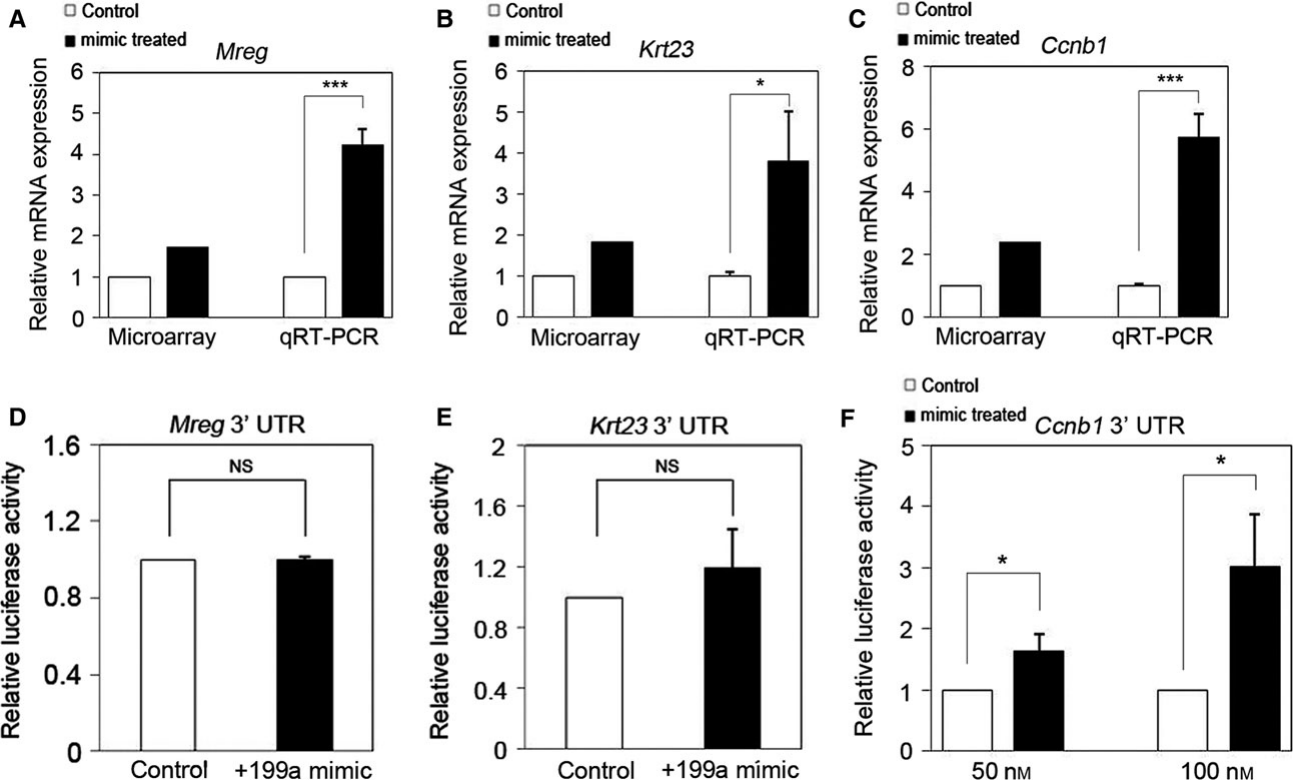
*Ccnb1* is a direct target gene of miR‐199a‐5p in mouse keratinocytes. (A–C) Validation of our previous microarray results using qRT‐PCR. Expression of (A) *Mreg*, (B) *Krt23*, and (C) *Ccnb1* was increased in PAM212 cells overexpressing miR‐199a in miR‐199a‐5p. The data were normalized against *GAPDH* mRNA expression. Results are the average of three independent experiments conducted in duplicate. (D–F) Results of dual luciferase reporter assays with constructs containing full‐length (D) *Mreg*, (E) *Krt23*, or (F) *Ccnb1* 3′ UTR mRNA expressed in PAM212 cells. Among the constructs tested, luciferase activity was significantly increased only in miR‐199a‐5p‐overexpessing cells cotransfected with the *Ccnb1* construct. *P < 0.05; ***P < 0.001. NS, not significant.

We have previously found that the expression of 232 genes was increased in PAM212 cells overexpressing miR‐199a‐5p (> 1.5‐fold, *P* < 0.05) 12. Among these genes, we selected *Ccnb1* and the genes encoding *Mreg* and *Krt23*, because they are also associated with skin keratinocytes 14, 15, 16. We validated the expression of these genes by qRT‐PCR using total RNA originally used as templates for the mRNA microarray analysis and found that the expression of *Mreg*,* Krt23*, and *Ccnb1* mRNA in PAM212 cells overexpressing miR‐199a‐5p was 3–5 times that in control cells (Fig. 1A–C). To determine whether the up‐regulation of these genes is due to direct targeting by miR‐199a‐5p, we used a luciferase assay system. While miR‐199a‐5p transfection did not affect the luciferase activity of *Mreg* and *Krt23* reporters (Fig. 1D–F), it increased the luciferase activity of the reporter containing the *Ccnb1* 3′ UTR in comparison with cells transfected with the control miR (Fig. 1F). These results suggested that *Ccnb1* is a target of miR‐199a‐5p in mouse keratinocytes.

### Mir‐199a‐5p directly up‐regulates *Ccnb1* expression in mouse keratinocyte

**Figure 2:**
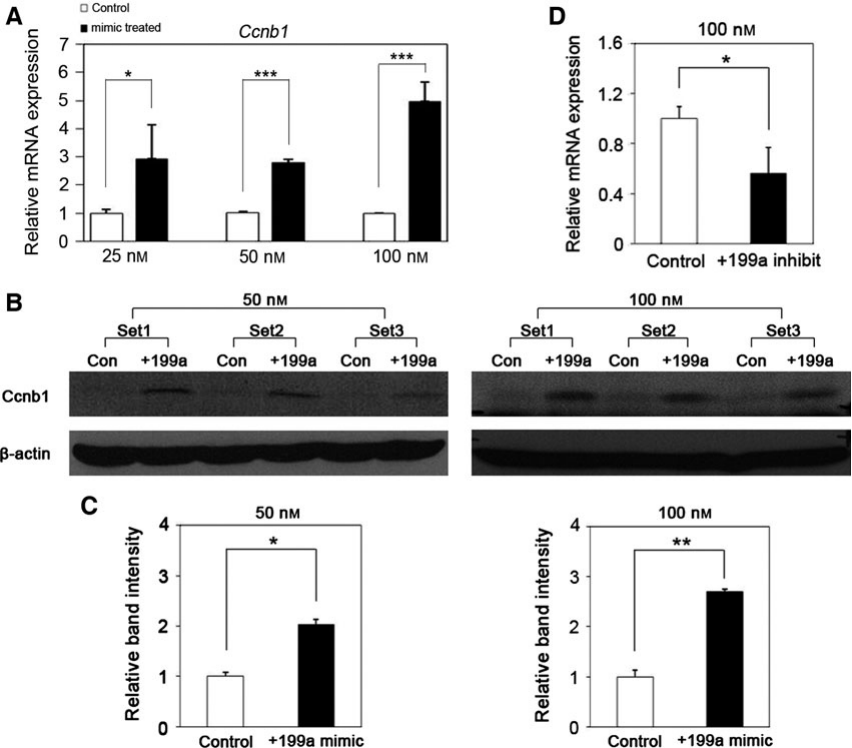
Expression of endogenous *Ccnb1* is increased by miR‐199a‐5p both at the mRNA and protein levels. (A) miR‐199a‐5p up‐regulated *Ccnb1* mRNA expression in PAM212 cells transfected with 25, 50, or 100 nm mimic. The data were normalized against GAPDH expression. Results are the average of three independent experiments conducted in duplicate. (B) Western blot analysis showed that the level of the CCNB1 protein was increased in PAM212 cells transfected with 50 or 100 nm miR‐199a‐5p mimic. β‐Actin was used as a loading control. (C) Quantitative analysis of western blots using imagej software (http://imagej.nih.gov/ij/index.html). The data were normalized against β‐actin expression. (D) Ccnb1 expression was significantly decreased by miR‐199a‐5p inhibitors. *P < 0.05; **P < 0.01; ***P < 0.001.

To investigate whether *Ccnb1* is a direct target of miR‐199a‐5p in mouse keratinocytes, we determined the expression of *Ccnb1* in miR‐199a‐5p‐overexpressing PAM212 cells at both mRNA and protein levels. qRT‐PCR revealed that *Ccnb1* mRNA expression was consistently higher in PAM212 cells transfected with miR‐199a‐5p than in cells transfected with the control RNA (Fig. 2A). Western blot analysis showed that CCNB1 expression was also increased in miR‐199a‐5p‐overexpressing PAM212 cells at both concentrations of the mimic (Fig. 2B). Overexpression of miR‐199a‐5p resulted in up‐regulated CCNB1 expression by 2.02‐ and 2.70‐folds at 50 and 100 nm mimic treatment, respectively (Fig. 2C). To further confirm these findings, we performed an inhibition experiment. Inhibition of endogenous miR‐199a‐5p expression using a miR‐199a‐5p inhibitor reduced the *Ccnb1* mRNA expression (Fig. 2D).

**Figure 3:**
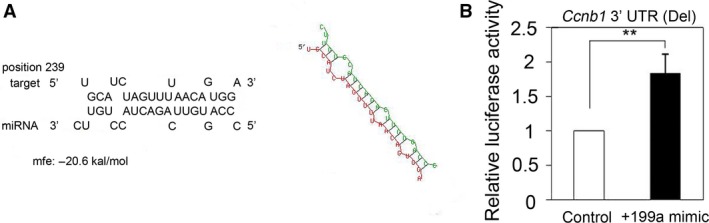
Investigation of miR‐199a‐5p‐binding site in *Ccnb1* 3′ UTR. (A) Prediction of miR‐199a‐5p‐binding site in *Ccnb1* 3′ UTR using RNAhybrid. (B) Luciferase assay was revealed that deletion of prediction site in *Ccnb1* 3′ UTR was not affected by miR‐199a‐5p. Results are the average of three independent experiments conducted in duplicate. **P < 0.01.

Next, we used several online software programs MIRBASE TARGETS (www.mirbase.org), TARGETSCAN (www.targetscan.org), MicroRNA.org (www.MicroRNA.org), and RNAHYBRID (bibiserv.techfak.uni-bielefeld.de/rnahybrid) to predict the target site of miR‐199a‐5p in *Ccnb1* mRNA. Only rnahybrid predicted a miR‐199a‐5p‐binding site at 239 bp of the *Ccnb1* 3′ UTR (Fig. 3A). To determine whether the predicted site is functional, we performed luciferase assay using a deletion mutant construct lacking this site. By comparing luciferase activity of the wild‐type and deletion constructs (Fig. 1F), we did not find any inhibitory effect of the deletion (Fig. 3B). These results indicated that the predicted site is not responsible for the miR‐199a‐5p‐dependent increase in CCNB1 expression, thus suggesting the presence of another site. Overall, the above results indicated that miR‐199a‐5p positively regulates *Ccnb1* expression at the post‐transcriptional level.

### Correlation between expressions of *Ccnb1* and miR‐199a‐5p in mouse hair cycle

**Figure 4:**
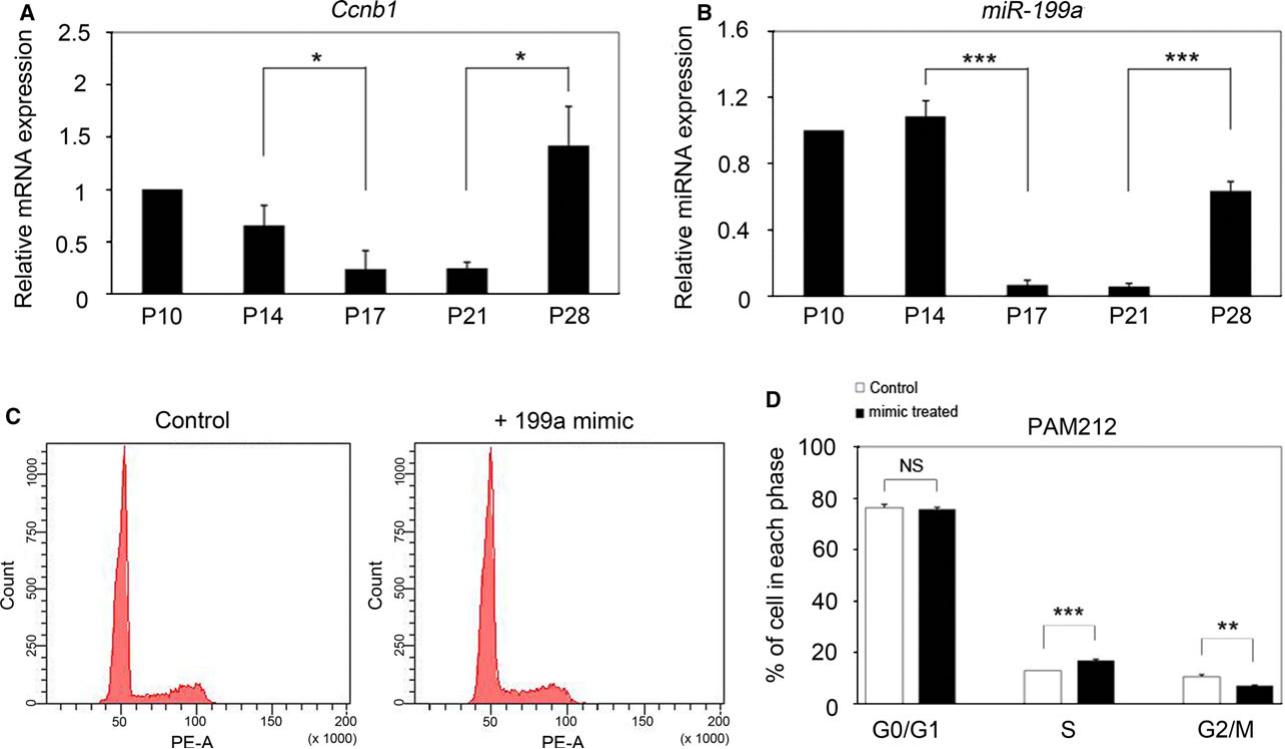
Expression of Ccnb1 during hair cycle and affection of cell cycle. (A, B) Relative expression of *Ccnb1* mRNA and miR‐199a‐5p during the hair cycle measured by qRT‐PCR. Both *Ccnb1* and miR‐199a‐5p were highly expressed during the anagen phases, and their expression decreased at telogen. The data were normalized against *GAPDH* mRNA expression. Results are the average of skin RNA isolated from skin of three mice; experiments were conducted in duplicate. (C, D) Overexpression of miR‐199a‐5p slightly affected the S and G2/M phases of cell cycle in PAM212 cells. *P < 0.05; **P < 0.01; ***P < 0.001.

We investigated whether the up‐regulation of *Ccnb1* by miR‐199a‐5p occurs during the mouse hair cycle. First, the relative expression of *Ccnb1* and miR‐199a‐5p was investigated at various stages of the hair cycle (P10–P28). qRT‐PCR analysis revealed that the expression of both *Ccnb1* and miR‐199a‐5p increased during the anagen phases and decreased at the following stages, with the lowest expression at telogen (Fig. 4A, B). These results showed that *Ccnb1* expression is positively correlated with miR‐199a‐5p expression in the mouse hair cycle.

Since CCNB1 plays a role in the cell cycle as a mitotic cyclin that functions in the G2/M phase transition, we performed cell cycle assay to determine whether increased miR‐199a‐5p expression affects the cell cycle in PAM212 cells. We found that the number of cells in S phase slightly increased and the number of cells in G2/M phase decreased after transfection with the miR‐199‐5p mimic in comparison with transfection with the negative mimic (Fig. 4C, D).

### *Ccnb1* expression is up‐regulated by miR‐199a‐5p in a mouse‐specific manner

**Figure 5:**
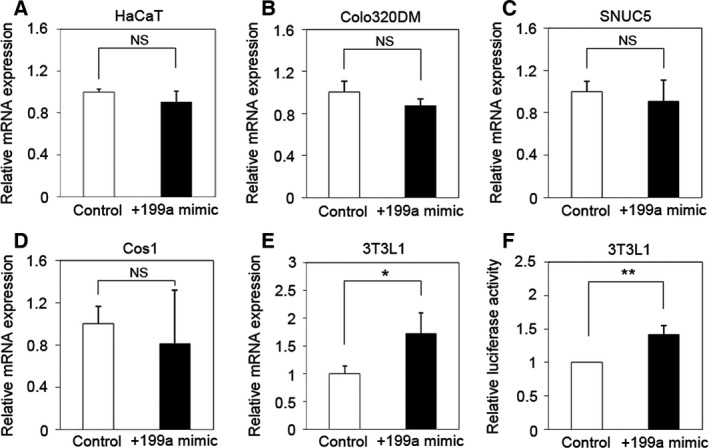
Up‐regulation of Ccnb1 by miR‐199a‐5p is mouse species‐specific. (A–E) qRT‐PCR revealed that relative *CCNB1* expression was not affected by miR‐199a‐5p overexpression in (A) HaCaT cells, (B) Colo320 DM, (C) SNU‐C5, and (D) Cos‐1 cells, whereas miR‐199a‐5p overexpression increased *Ccnb1* expression in (E) 3T3‐L1 cells. The data were normalized against GAPDH mRNA expression. Results are the average of three independent experiments conducted in duplicate. (F) luciferase activity of *Ccnb1* 3′‐UTR was also significantly increased by miR‐199a‐5p overexpression in 3T3‐L1 cells. *P < 0.05; **P < 0.01; NS, not significant.

Next, we investigated whether *Ccnb1* expression was also up‐regulated by miR‐199a‐5p in the immortalized human keratinocyte cell line HaCaT. In contrast to PAM212 cells, we found that the *CCNB1* mRNA expression level was not affected by miR‐199a‐5p overexpression in HaCaT cells (Fig. 5A). To confirm this finding, we investigated the effect of miR‐199a‐5p overexpression on *CCNB1* expression in other primate cell lines. We found that *CCNB1* expression was not affected in the human colorectal cell lines SNU‐C5 and Colo320 DM (Fig. 5B, C) and in the monkey fibroblast cell line Cos‐1 (Fig. 5D). Interestingly, miR‐199a‐5p overexpression increased the expression of *Ccnb1* in another mouse cell line, 3T3‐L1, similar to the effect in PAM212 cells (Fig. 5E). Furthermore, the *Ccnb1* 3′ UTR responded to the miR‐199a‐5p mimic, as shown by the increased luciferase activity in 3T3‐L1 cells (Fig. 5F). These data suggest that increased expression of *Ccnb1* in response to miR‐199a‐5p is a mouse‐specific phenomenon.

**Figure 6:**
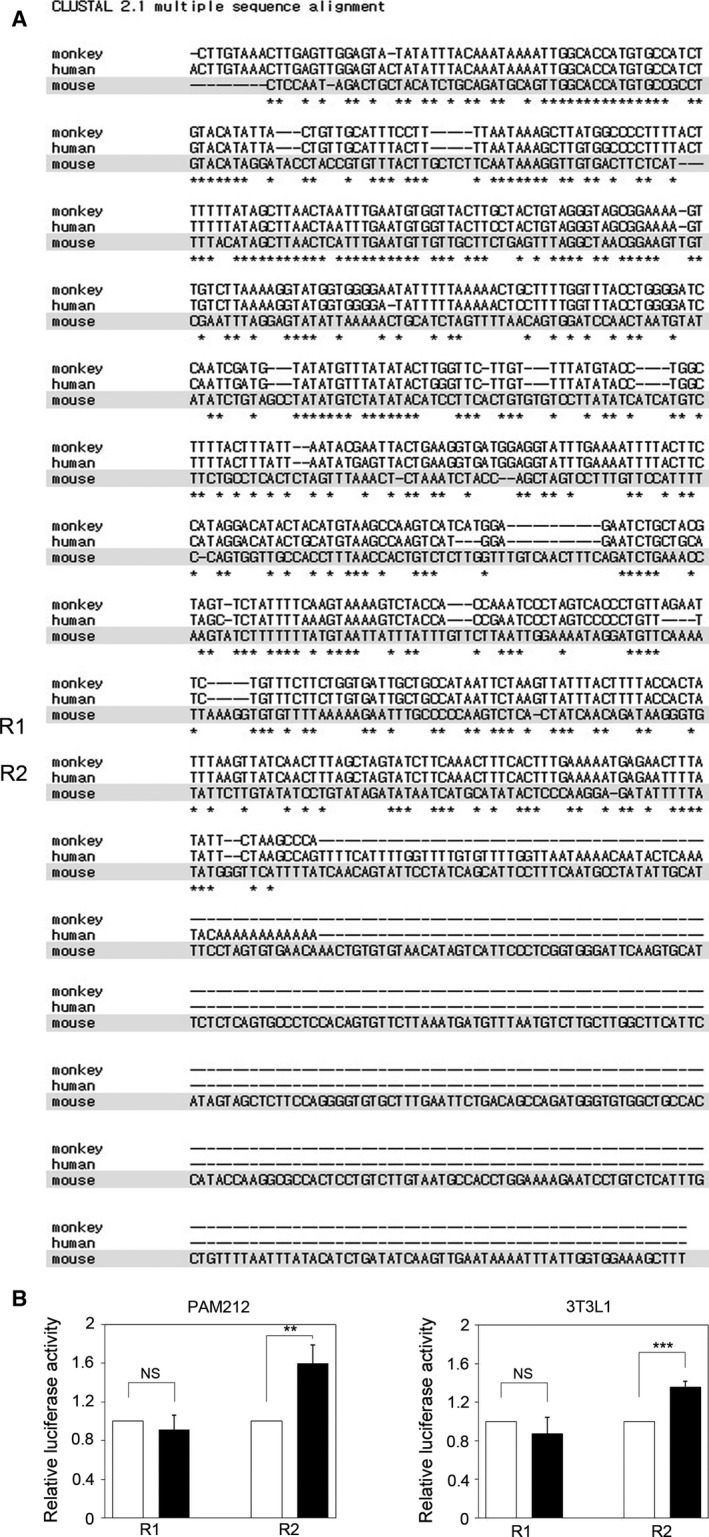
Identification of the *Ccnb1* 3′ UTR region responsible for miR‐199a‐5p‐induced regulation of expression in the mouse. (A) Sequence alignment of *Ccnb1* 3′ UTRs from monkey, human, and mouse; the alignment was generated using clustalw (www.genome.jp/tools/clustalw). (B, C) Dual luciferase reporter assays with constructs containing the R1 or R2 region of the *Ccnb1* 3′‐UTR in (B) PAM212 and (C) 3T3‐L1 cells. In both cell types, miR‐199a‐5p only activated luciferase activity of the R2 region. In contrast, the R1 region was not regulated by miR‐199a‐5p. Results are the average of three independent experiments conducted in duplicate. **P < 0.01; ***P < 0.001; NS, not significant.

To analyze whether this differential regulation is caused by structural differences in the *Ccnb1* 3′ UTR, we examined the alignment of mouse, monkey, and human 3′ UTR sequences. Interestingly, we found that the mouse *Ccnb1* 3′ UTR (939 bp) is much longer than those of human (622 bp) or monkey (569 bp) (Fig. 6A). This difference is also present in between the mouse and other species. Therefore, we hypothesized that mouse‐specific regulation of *Ccnb1* expression by miR‐199a‐5p depends on the mouse‐specific 3′ UTR region. To verify this hypothesis, we compared luciferase activities of the 3′ UTR constructs containing either the evolutionarily conserved region of the 3′ UTR (R1; 1–513 bp) or the mouse‐specific region (R2; 491–939 bp). As expected, we found that luciferase activity of the R2‐containing construct was significantly increased by miR‐199a‐5p in both PAM212 and 3T3‐L1cells, while that of the R1‐containing construct was not (Fig. 6B, C). Moreover, the increase in luciferase activity by miR‐199a‐5p conferred by the R2 region was similar to that of the full‐length *Ccnb1* 3′ UTR. From these results, we conclude that the up‐regulation of *Ccnb1* by miR‐199a‐5p is mediated by the mouse‐specific region of the *Ccnb1* 3′ UTR.

## Discussion

In general, the function of miRNA is to inhibit gene expression at the post‐transcriptional level by binding to the 3′ UTRs of specific target mRNA 2. However, several studies have demonstrated that miRNA are also able to post‐transcriptionally up‐regulate their target genes. For instance, miR‐466l increases IL‐10 expression in Toll‐like receptor‐triggered macrophages by antagonizing the interaction between the RNA‐binding protein tristetraprolin and IL‐10 mRNA 17. A microRNA, miR‐145 promotes vascular smooth muscle cell differentiation in part by increasing myocardin protein expression 18. Overexpression of miR‐223 increases the total cellular level of glucose transporter type 4 protein in neonatal rat ventricular myocytes 19.

In this study, we showed that miR‐199a‐5p up‐regulates CCNB1 expression in mouse keratinocytes and fibroblasts. Up‐regulation of *Ccnb1* by miRNA has been previously reported. Huang *et al*. 20 demonstrated that miR‐744, miR‐1186, and miR‐466d‐3p induce *Ccnb1* expression by interacting with its promoter region in mouse cell lines. In contrast, we found that *Ccnb1* up‐regulation by miR‐199a‐5p is mediated through the 3′ UTR of *Ccnb1*. Although we did not identify the precise binding site of miR‐199a‐5p in the *Ccnb1* 3′ UTR because *in silico* sequence analysis showed no predicted miR‐199a‐5p target sites, dual luciferase assay suggested that the 3′ UTR of *Ccnb1* is a direct target of miR‐199a‐5p in mouse keratinocytes and fibroblasts.

We also found that the up‐regulation of *Ccnb1* expression by miR‐199a‐5p is mouse specific. Our data show that miR‐199a‐5p overexpression did not affect *Ccnb1* expression in human and monkey cells. This was unexpected because most miRNA‐binding sites on mRNA are conserved between species. However, there are some nonconserved miRNA‐binding sites that cause species‐specific miRNA–mRNA interactions. For instance, miR‐351 and miR‐298 regulate astrocyte activation by targeting genes involved in the tumor necrosis factor‐alpha (TNF‐α) signaling pathway in a mouse‐ and rat‐specific manner 21. FOXO1 regulates cell proliferation and invasion via miR‐183 only in human cells 22. These data suggest that changes in miRNA‐mediated regulation of target genes occurred in a species‐specific manner and contributed to phenotypic differences among various species.

**Figure 7:**
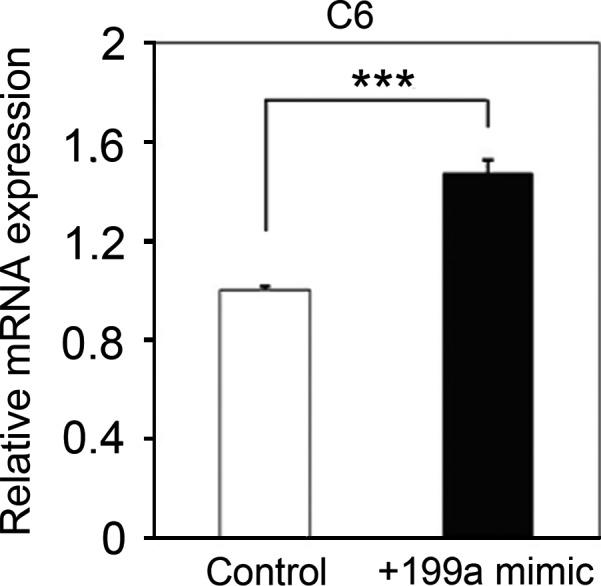
The effect of miR‐199a‐5p to *CCNB1* expression in rat C6 cell. qRT‐PCR revealed that *CCNB1* expression was increased by miR‐199a‐5p in rat glial cell. Results are the average of three independent experiments. Results are the average of three independent experiments conducted in duplicate. ***P < 0.001.

Using a reporter assay, we demonstrated that miR‐199a‐5p up‐regulates *Ccnb1* expression by binding specific sequences in the mouse *Ccnb1* 3′ UTR. There is a sequence variation in the mouse *Ccnb1* 3′ UTR compared with those of other species. We found that the expression of rat *Ccnb1*, which has a long 3′ UTR similar to that of mouse *Ccnb1*, was increased by miR‐199a‐5p (Fig. 7). Interestingly, hamster, another rodent, has a *Ccnb1* 3′ UTR of only 684 bp. Mouse and rat belong to the Muridae family, whereas hamster belongs to the Cricetidae. It would be interesting to see whether the regulation of CCNB1 by miR‐199a‐5p is present only in the Muridae or is common to all rodents. Further studies are required to determine the precise regulation mechanism.

**Figure 8:**
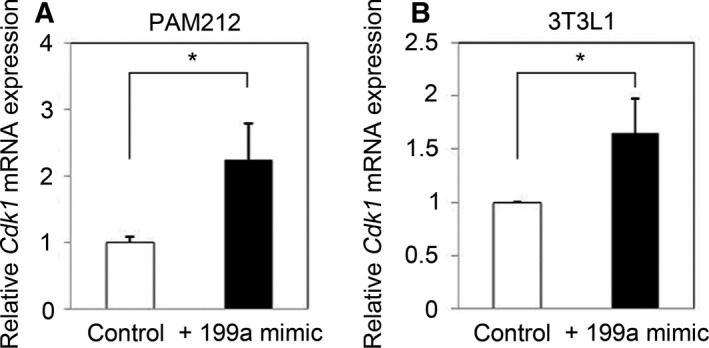
The effect of miR‐199a‐5p to *Cdk1* expression. qRT‐PCR revealed that Cdk1 expression was increased by miR‐199a‐5p in mouse keratinocyte (A) and fibroblast (B). Results are the average of three independent experiments conducted in duplicate. *P < 0.05.

*Ccnb1* is well known to act in G2/M phase transition during the cell cycle 23, 24. It forms a complex with cyclin‐dependent kinase 1 (Cdk1), and this complex (maturation‐promoting factor) induces the early events of mitosis by controlling chromosome condensation, nuclear envelope breakdown, and spindle pole assembly. Interestingly, we also found that *Cdk1* expression is concomitantly increased by miR‐199a‐5p in mouse keratinocytes and fibroblasts (Fig. 8). On the basis of this information, we speculated that miR‐199a‐5p regulates the mouse keratinocyte cell cycle by up‐regulating the expression of *Ccnb1* and *Cdk1*. Unexpectedly, the up‐regulation of *Ccnb1* by miR‐199a‐5p did not markedly affect cell cycle phases in mouse keratinocytes (Fig. 3D). Thus, these data suggest that the increased expression of *Ccnb1* and *Cdk1* is not sufficient to change cell cycle. Alternatively, the up‐regulation of *Ccnb*1 and *Cdk1* expression by miR‐199a‐5p may play other, yet unidentified roles in mouse keratinocytes. This may be more plausible than a role in cell cycle, because our previous study showed that increased miR‐199a‐5p expression does not induce proliferation in PAM212 cells 12. The precise role of *Ccnb1*/*Cdk1* up‐regulation by miR‐199a‐5p in mouse keratinocytes is unclear. Functional annotation analysis revealed that expression of the genes involved in not only cell cycle (50 genes) but also in cell division process (34 genes) was changed by miR‐199a‐5p in PAM212 cells (Table 2). This may have resulted in the combined effect of no proliferation of cells with the up‐regulation of *Ccnb1*. Additional study is necessary to elucidate the mechanism and effect of this regulation in mouse cells.

**Table 2:** List of genes that involved in cell cycle and cell division process

Cell cycle (50 genes)
*CLSPN, KIFC1, PRC1, KNTC1, AURKA, AURKB, CDT1, CDCA8, MCM7, INCENP, CDCA2, H2AFX, CDCA5, CDCA3, CDC7, CDC6, KIF11, LIG1, SGOL2, SGOL1, NUSAP1, ESPL1, MCM2, RB1, MCM3, ESCO2, NCAPD3, NCAPD2, MCM6, RAD51, UHRF1, FANCD2, SPAG5, BUB1B, TIPIN, ANLN, SPC25, NCAPH, TRP53INP1, TFDP1, CKAP2, MKI67, NDC80, BIRC5, CDC25C, GSG2, 2610039C10RIK, CCNB1, KIF20B, CHAF1A*
Cell division (34 genes)
*KIFC1, PRC1, TIPIN, KNTC1, ANLN, AURKB, SPC25, CDCA8, NCAPH, CDCA7, INCENP, CDCA2, CDCA5, TOP2A, CDCA3, CDC7, CDC6, KIF11, SGOL2, LIG1, SGOL1, NUSAP1, BIRC5, NDC80, RB1, CDC25C, NCAPD3, MCM5, 2610039C10RIK, NCAPD2, CCNB1, SPAG5, KIF20B, BUB1B*

In addition, miR‐199a‐5p and miR‐199b‐5p can potentially regulate the same transcripts because they have identical seed sequence. Since expression of miR‐199b‐5p in skin keratinocyte or hair follicle has not been documented, further studies are required to address this question.

In conclusion, our data indicate that *Ccnb1* expression is increased by miR‐199a‐5p in a mouse‐specific manner. Although further studies are required to understand the roles of miR‐199a‐5p and *Ccnb1*, these results reveal a new evolutionary relationship between *Ccnb1* and miR‐199a‐5p in mouse keratinocytes and thus make a contribution to miRNA biology.

## Conclusions

MiR‐199a‐5p up‐regulates *Ccnb1* expression in a mouse‐specific manner. These results indicate an evolutionary relationship between *Ccnb1* and miR‐199a‐5p in mouse keratinocytes.

## Author contributions

BKK and SKY designed the experiments. BKK, IK, ARL, and HIY conducted the experiments. BKK and SKY wrote the paper.

## Supporting information


**Table S1.** Sequence alignment of Ccnb1 3′ UTR among various species using clustalw.
