# Supplementary material for: Mouse‐specific up‐regulation of Ccnb1 expression by miR‐199a‐5p in keratinocyte
Source: FEBS Open Bio. 2016 Oct 24;6(11):1131–40. doi: 10.1002/2211-5463.12133 (PMC5095150; doi:10.1002/2211-5463.12133)
Supplement: Supplementary file 1 — Table S1. Sequence alignment of Ccnb1 3′ UTR among various species using clustalw. [file FEB4-6-1131-s001.doc]

**Table S1. Sequence alignment of Ccnb1 3’UTR among various species using CLUSTALW**

Sequence 1: monkey 569 bp

Sequence 2: human 623 bp

Sequence 3: pig 600 bp

Sequence 4: cow 111 bp

Sequence 5: mouse 939 bp

Sequence 6: rat 1901 bp

Sequence 7: hamster 684 bp

Sequence 8: zebrafish 204 bp

mouse ---------CTCCAAT-AGACTGCTACA-----TCTGCAGATGCAGTTGGC-ACCATGTG

rat --------ACTCGAAT-GGACTACCTCG-----TCTGCAGATGCAGTTGGC-ACCATGTG

hamster --------ACTCAAGT-GGACCACTACACTACATCGGCAAATGCAATTGGC-ACCATGTG

monkey -CTTGTAAACTTGAGTTGGAGTA-TATA-----TTTACAAATAAAATTGGC-ACCATGTG

human ACTTGTAAACTTGAGTTGGAGTACTATA-----TTTACAAATAAAATTGGC-ACCATGTG

pig -CTTGTGAACTTCGGA-ATACTATAATA-----TCTACAAATAAAATTGGC-ACCATGTG

cow --TTGTGAACTTTGGA-ATACTAC----------CTGCAAATAAAATTGGC-ACCATGTG

zebrafish --------------TTGGGGTTA-----------TGCTGAAGAGACTTAACGACTGTGTG

* * ** * ** ****

mouse CCGCCT-GTACATAGGATACCTACCGTGTTTACTTGCTCTTCAATAAAGGTTGTGA-CTT

rat CCGCCT-GTACATAGGAT----ACCATGTTTATTTGCTCTTCAATAAAGGTTGTGA-CTT

hamster CCACCT-GTACATAGTAT----ACAGTGTTTACTTGCTCTTCAATAAAGGCTGT------

monkey CCATCT-GTACATATTAC---TGTTGCA----TTTCCTTTT-AATAAAGCTTATGGCCCC

human CCATCT-GTACATATTAC---TGTTGCA----TTTACTTTT-AATAAAGCTTGTGGCCCC

pig CCATCT-GTACATATTATA--TGTTACACTTATTTACTTTT-AATAAAGTTTGTAGTCCT

cow CCATCT-GTACATATTATA--TGTTACATTTATTTACTTTT-AATAAAGTT-GAAGTTCT

zebrafish CTGCCTTGTAAAAATTGTAACTTATGCATGT-TTTACTGTT----AAACTTTTTATTGCA

* ** *** * * ** ** ** ***

mouse CTC-ATTTTAC--ATAGCTTAACTCATTTGAATGTTGTTGCTTCTGAGTTTAGGCTAAC-

rat TTTTACTTTGC--ATAGCTCAACTCATTTGAATGTGGTTGCTTCTGACTTTAGGATAAC-

hamster -TTTACTTTTC--ATAGCTTACCTCATTTGAATGTTGGTTGTTCTGACTCTAGGATAAC-

monkey TTTTACTTTTTT-ATAGCTTAACTAATTTGAATGTGGTTACTTGCTACTGTAGGGTAGCG

human TTTTACTTTTTT-ATAGCTTAACTAATTTGAATGTGGTTACTTCCTACTGTAGGGTAGCG

pig TTTTACTT---------CTTAACTCATTTGAATGTGGCTATTTCCCACTTGAGGATAACT

cow TTTTACTT---------AA-----------------------------------------

zebrafish ATTTATTTTACGGATTTCTTCACTGCCATGGGT-TGAGCAAAAGTAATCTTTTAATGATT

* * **

mouse -GGAAGTTGTC---GA------ATTTAGGAGTATATTA---------AAAACTGCATCTA

rat -AGAAGTTGTCAAAGA------ATTTAGGAGTATATCA---------AAAACTGCTTCTA

hamster -AAGGGTTGTCTAAAA-----CATTTAGGATTATTTTT---------GAAACTGCTTTTA

monkey GAAAAGTTGTCTTAAAAGGTATGGTGGGGAATATTTTT--------AAAAACTGCTTTTG

human GAAAAGTTGTCTTAAAAGGTATGGTGGGGA-TATTTTT--------AAAAACTCCTTTTG

pig TAAAAGTTGTCTTAAA-GGTACAGTGGAGAATGTTTTTTAAAAAATGAAAACTGTTTTCA

cow ------------------------------------------------------------

zebrafish CAAAGTGTTTATGCAAGTTTGTACATTTGAATTCAAAT----------AAACTTTTTAAA

mouse GTTTTAACAGTGGATCCAACTAATGTATATATCTGTAGCCTATATGTCTATAT-ACATCC

rat GTTT-AACAGTGGATTCAGTTAATGTATATATGTGTAGCCTATAT-TTTATAT-ACTTGC

hamster GTTT-AACAGAGAATCCAAGTAATGTACGTAATTGTAGCCTATATGTTTATGT-ACTTCC

monkey GTTT-ACCTGGGGATCCAATCGATGTATATGTTTATATACTTGGTTCTTGTTTTATGTAC

human GTTT-ACCTGGGGATCCAATTGATGTATATGTTTATATACTGGGTTCTTGTTTTATATAC

pig GTT--ACCTGGGAACCCAACTAATATATACAATTGGCTC-----TTCTTGTTTTATGTAC

cow ------------------------------------------------------------

zebrafish GTTTT-------------------------------------------------------

mouse TTCACTG-TGTGTCCT---TATATCATC--------------ATGTCTTCTGCCTCACTC

rat TTCACTT-TGTGTGTC---CTTATCATG--------------TAGTCATCTGCCTAGCTC

hamster TTCTTTAATGTGTCCTGGTCATATCTTTTAAGTCATCCTGCAAAGCCCTCTGCCTAGCTC

monkey CTGGCTTTTACTTTATTAATACGAATTACTG----------AAGGTGATGGAGGTATTTG

human CTGGCTTTTACTTTATTAATATGAGTTACTG----------AAGGTGATGGAGGTATTTG

pig TTGGCATA-ACTTAATTAATATGAGTTCAT-----------ATAGTCTTGAAGCCATTTA

cow ------------------------------------------------------------

zebrafish ------------------------------------------------------------

mouse TAGTTTAAACTCTAAATCTACCAGCTAGTCCTTTGTTCCATTTTCCAGT------GGTTG

rat TGGGTTAAACTCCAAGTCTACCAGCTAGTCCT---------------------------G

hamster TTGTTTAAACTGTCAATTTACCAGCTTGTCCTTAGTTCCCTTTTCTATTTCTTCAGGTGG

monkey AAAATTTTACTTCCATAGGACATACTACATGTAAGCCAAGTCATCATG---GAGAATCTG

human AAAATTTTACTTCCATAGGACATACTGCATGTAAGCCAAGTCAT---G---GAGAATCTG

pig A------TATCTTTATATGTTACACTGTATGTAAGCTCAGTCATCTTGA--GAGAATCTG

cow ------------------------------------------------------------

zebrafish ------------------------------------------------------------

mouse CCACCTTTAACCACTGTCTCTTGGTTTGTCAACTTTCAGATCT-GAAACCAAGTATC-TT

rat CTGCCTGAAACCACTGTCTCTTGAGCTGTCAACTTTCAGATCTTGAAACGAAGTGT--TT

hamster TTGCTGTCCTTCACTGTGTCTTGAGCTATGGACTTTCAGATCT-GAACCCCAGT-----T

monkey CTACGTAGTTCTATTTTCAAGTAAAA-GTCTACCACCAAATCCCTAGTCACCCTGTTAGA

human CTGCATAGCTCTATTTTAAAGTAAAA-GTCTACCACCGAATCCCTAGTCCCCCTGTT---

pig CTACCTAGTTCTAC--ACAAGGAAGA-GTCTACCGTCTCAATCCTAGTCCCCTTGTT---

cow ------------------------------------------------------------

zebrafish ------------------------------------------------------------

mouse TTTTTATGTAATTATTTATTTGTT--CTTAATTGGAAAATAGG-ATGTTCAAAATTA---

rat TTGTTATGTAATTATTTATTTGTT--CTTAATTGGAAGGTAGG-ATGTTCAAAATTA---

hamster TTCTTGTGTAATTATTTATTTGTTAATTGGCTTGGGAAATAGC-ATGTTTAAAATTA---

monkey ATTCTGTTTCTTCTGGTGATTGCTGCCATAATTCTAAGTTATTTACTTTTACCACTAT-T

human -TTCTGTTTCTTCTTGTGATTGCTGCCATAATTCTAAGTTATTTACTTTTACCACTAT-T

pig -TTATATTTCCTCTGGTGGCTGCAGTCATAATCCTAAATAATCTACTTGTACCACTTTCT

cow ------------------------------------------------------------

zebrafish ------------------------------------------------------------

mouse -AAGGTGTGTTTTAAAAAGAATTTGCCCCCAAGTCTCACTATCAACAGATAAGGGTGTAT

rat -AAGGTGTCTT---AAAATAATTTGCCCCTAAGTCTCAGTACCAACAGATAAGGGTGTAT

hamster -AAGGTATA-----AAAAGAATTTGCCCCTTAATCTCA----------------------

monkey TAAGTTATCAACT-TTAGCTAGTATCTTCAAACTTTCACT-TTGAAAAATGAGAACTTTA

human TAAGTTATCAACT-TTAGCTAGTATCTTCAAACTTTCACT-TTGAAAAATGAGAATTTTA

pig TAAATTATCAACT-TTAG-TATCAACTT-----TTTCACT-TGGAAAAATGAGAATTTTA

cow ------------------------------------------------------------

zebrafish ------------------------------------------------------------

mouse TCTTGTATATCCTGTATAGATATAATCATGCATATACTCCCAAGGAGATATTTTTATATG

rat TCTTGTATATCCTGTATAGATATAATCATGCATTTATTGTCGAGGAGATGTTTTTATGTG

hamster ------ATGTCCTATATAAATGTAATCATGCATATGTTGTCAAGGAGATA--------TG

monkey TATTCTAAGCCCA-----------------------------------------------

human TATTCTAAGCCAGTTTTCATTTTGGTTTTGTGTTTTGGTTAATAAAACAATACTCAAATA

pig ATTTATATTCAAAACCTAATTTACTTTTTGTTTATTGGTTAAGAAAAATAAAACAATCCT

cow ------------------------------------------------------------

zebrafish ------------------------------------------------------------

mouse GGTTCATTTT-ATCAACAGTATTCCTATCAGCATTCCTTTCAATGCCTATATTGCATTTC

rat GGTTCATTT--ATCAACAGTAATCCTTTTAGCATCC--TTCAAGGCCTATATTGCATTTC

hamster GACTCATTTTTTTCAACAATATTCCT------------TTTAATGCCTATATTGCATTTC

monkey ------------------------------------------------------------

human CAAAAAAAAAAAA-----------------------------------------------

pig TAGA--------------------------------------------------------

cow ------------------------------------------------------------

zebrafish ------------------------------------------------------------

mouse CTA-GTGTGAA---CAAACTGTGTGTAACA-----TAGTCATTCCCTCGGTGGGATTCAA

rat CTA-GTGTGAA---TAAACGATGTAATATAGTAGTCAATCGTTCCCTTAGTGATATTCAA

hamster CTAAGTGTACATTTCATACTGTATGTAATG-TATTCAGTGATCCTTTACAATACATTCAA

monkey ------------------------------------------------------------

human ------------------------------------------------------------

pig ------------------------------------------------------------

cow ------------------------------------------------------------

zebrafish ------------------------------------------------------------

mouse GT-GCATTCTCTCAGTGCC-CTCCACAGTGTTCTTAAATGATGTTTAATGTCTTGCTTGG

rat GT-GCATTCTCTCACTGCCTCTCCACAATGCTGCTTAACACTGTTTAATACCTTGCCTGG

hamster ATTGCATTCTC-------------------------------------------------

monkey ------------------------------------------------------------

human ------------------------------------------------------------

pig ------------------------------------------------------------

cow ------------------------------------------------------------

zebrafish ------------------------------------------------------------

mouse CTTCATTCATAGTAGCTCTTCCAGGGG-TGTGCTTTGAATTCTGACAGCCAGATGGGTGT

rat CTCCATGCATAGTAGCTCTCCCAGGGGGTGTGCTTTCAATTCTGACAGCCAGATGAGTGC

hamster ------------------------------------------------------------

monkey ------------------------------------------------------------

human ------------------------------------------------------------

pig ------------------------------------------------------------

cow ------------------------------------------------------------

zebrafish ------------------------------------------------------------

mouse GGCTGCCACCATACCAAGGCGCCACT-------CCTGTCTTGTAATGCCACCTGGAAAAG

rat GGCTGGCACCATGCCAAGGTGCCACTTCTTTCTCCTGTTTTGTAACACCAACTGGAAAAA

hamster ------------------------------------------------------------

monkey ------------------------------------------------------------

human ------------------------------------------------------------

pig ------------------------------------------------------------

cow ------------------------------------------------------------

zebrafish ------------------------------------------------------------

mouse AATCCTGTCTCATTTGCTGTTTTAATTTATACATCTGATATCAAGTTGAATAAAATT-TA

rat AATCCTGTCTCATTTGCTGTTTTAATTTATACATCTGATTCCGAGTTGAATAAAATTGTA

hamster ------------------------------------------------------------

monkey ------------------------------------------------------------

human ------------------------------------------------------------

pig ------------------------------------------------------------

cow ------------------------------------------------------------

zebrafish ------------------------------------------------------------

mouse TTGGTGGAAAGCTTT---------------------------------------------

rat TTTCTGGAAAGCTTTCTTTCCCATTCACATTTGCCCTCTTGACTGTGTGTTGTCTTTTGC

hamster ------------------------------------------------------------

monkey ------------------------------------------------------------

human ------------------------------------------------------------

pig ------------------------------------------------------------

cow ------------------------------------------------------------

zebrafish ------------------------------------------------------------

mouse ------------------------------------------------------------

rat TATATGGAACCTTCTGTTGCTAACAAACATCTTCCTATAGTTCTAGATTTATTTTTTTTT

hamster ------------------------------------------------------------

monkey ------------------------------------------------------------

human ------------------------------------------------------------

pig ------------------------------------------------------------

cow ------------------------------------------------------------

zebrafish ------------------------------------------------------------

mouse ------------------------------------------------------------

rat TTATTCTTTTTTTTCTCGGAGCAGGGGACCGAACCCAGGGCCTTGTGCTTGCTAGGCAAG

hamster ------------------------------------------------------------

monkey ------------------------------------------------------------

human ------------------------------------------------------------

pig ------------------------------------------------------------

cow ------------------------------------------------------------

zebrafish ------------------------------------------------------------

mouse ------------------------------------------------------------

rat TGCTCTACCACTGAGCTAAATCCCCAACCCCCTAGATTTTCAATCATAAGGTGGGCCACA

hamster ------------------------------------------------------------

monkey ------------------------------------------------------------

human ------------------------------------------------------------

pig ------------------------------------------------------------

cow ------------------------------------------------------------

zebrafish ------------------------------------------------------------

mouse ------------------------------------------------------------

rat CCTTGTCCCAGCACAATGGAGGCAGAGACAGGTAGACTTTTTATGGGTTCAAGGCCAGAC

hamster ------------------------------------------------------------

monkey ------------------------------------------------------------

human ------------------------------------------------------------

pig ------------------------------------------------------------

cow ------------------------------------------------------------

zebrafish ------------------------------------------------------------

mouse ------------------------------------------------------------

rat CCTGTCTCAGAAAAAATTTTTTTCCAAAATGCTTTCTCTTAGGTGTACATTCCTTCCTTC

hamster ------------------------------------------------------------

monkey ------------------------------------------------------------

human ------------------------------------------------------------

pig ------------------------------------------------------------

cow ------------------------------------------------------------

zebrafish ------------------------------------------------------------

mouse ------------------------------------------------------------

rat GATGAGTAAAGTCTAAGTGTATGACGATGTACGATGTATGTCGGTGATTAAGTGTACATT

hamster ------------------------------------------------------------

monkey ------------------------------------------------------------

human ------------------------------------------------------------

pig ------------------------------------------------------------

cow ------------------------------------------------------------

zebrafish ------------------------------------------------------------

mouse ------------------------------------------------------------

rat TCTGGTCTAAATGTTGGTTTGCCCCTGTAATAATAGTTTTTAAAACCGAGTTCCAGAGTT

hamster ------------------------------------------------------------

monkey ------------------------------------------------------------

human ------------------------------------------------------------

pig ------------------------------------------------------------

cow ------------------------------------------------------------

zebrafish ------------------------------------------------------------

mouse ------------------------------------------------------------

rat TTTGTGGCCACCTACTATGGCTTGACTGTTCTCAAGTACTTGGCATTGTCAAACATTTGC

hamster ------------------------------------------------------------

monkey ------------------------------------------------------------

human ------------------------------------------------------------

pig ------------------------------------------------------------

cow ------------------------------------------------------------

zebrafish ------------------------------------------------------------

mouse ------------------------------------------------------------

rat CATATGAACTTGAACAGTGTTCAGTTGCCAGTGCAAGGTTTTAACTGTTGTCTATTACAT

hamster ------------------------------------------------------------

monkey ------------------------------------------------------------

human ------------------------------------------------------------

pig ------------------------------------------------------------

cow ------------------------------------------------------------

zebrafish ------------------------------------------------------------

mouse ------------------------------------------------------------

rat CAGCTTGGTGTAGAGCTGCGTGTATCTTGTTATTTAGAAACTTCTTGTTGAGCCTTCACT

hamster ------------------------------------------------------------

monkey ------------------------------------------------------------

human ------------------------------------------------------------

pig ------------------------------------------------------------

cow ------------------------------------------------------------

zebrafish ------------------------------------------------------------

mouse ------------------------------------------------------------

rat GTACAGTCCTGAGGTTCCTTACTTATGCTGGGGTGAAAACTTGTGAAAAATCAACTTCTT

hamster ------------------------------------------------------------

monkey ------------------------------------------------------------

human ------------------------------------------------------------

pig ------------------------------------------------------------

cow ------------------------------------------------------------

zebrafish ------------------------------------------------------------

mouse ------------------------------------------------------------

rat TGAAATTAAATTAAAATCTAATTGTGTAATTTCCCCTTTACTTTTCTTCAGCCTCTCCCA

hamster ------------------------------------------------------------

monkey ------------------------------------------------------------

human ------------------------------------------------------------

pig ------------------------------------------------------------

cow ------------------------------------------------------------

zebrafish ------------------------------------------------------------

mouse ------------------------------------------------------------

rat TGGAACCCACCCTCCCCTTGCATTCTCAAGCTCATGACCTCTGGCTTTTGTTGATGTGAA

hamster ------------------------------------------------------------

monkey ------------------------------------------------------------

human ------------------------------------------------------------

pig ------------------------------------------------------------

cow ------------------------------------------------------------

zebrafish ------------------------------------------------------------

mouse ------------------------------------------------------------

rat TATGTTCATAAGTATATAATCGGCACAATATGTTGTATGTATTTCAGCACTGACCCCTGT

hamster ------------------------------------------------------------

monkey ------------------------------------------------------------

human ------------------------------------------------------------

pig ------------------------------------------------------------

cow ------------------------------------------------------------

zebrafish ------------------------------------------------------------

mouse ------------------------------------------------------------

rat TACCTGTAGTTGTCTTGGATTGCCTCCCACCCCCATGAGTTTCCCCTTCTGGGTTGATAT

hamster ------------------------------------------------------------

monkey ------------------------------------------------------------

human ------------------------------------------------------------

pig ------------------------------------------------------------

cow ------------------------------------------------------------

zebrafish ------------------------------------------------------------

mouse ------------------------------

rat CATCTTTATTAAAGTCCTGATTAAACTTTA

hamster ------------------------------

monkey ------------------------------

human ------------------------------

pig ------------------------------

cow ------------------------------

zebrafish ------------------------------
